# Auditory cortical field coding long-lasting tonal offsets in mice

**DOI:** 10.1038/srep34421

**Published:** 2016-09-30

**Authors:** Hironori Baba, Hiroaki Tsukano, Ryuichi Hishida, Kuniyuki Takahashi, Arata Horii, Sugata Takahashi, Katsuei Shibuki

**Affiliations:** 1 Department of Neurophysiology, Brain Research Institute, Niigata University, 1-757 Asahi-machi, Chuo-ku, Niigata 951-8585, Japan; 2 Department of Otolaryngology, Head and Neck Surgery, Graduate School of Medical and Dental Sciences, Niigata University, 1-757 Asahi-machi, Chuo-ku, Niigata 951-8510, Japan

## Abstract

Although temporal information processing is important in auditory perception, the mechanisms for coding tonal offsets are unknown. We investigated cortical responses elicited at the offset of tonal stimuli using flavoprotein fluorescence imaging in mice. Off-responses were clearly observed at the offset of tonal stimuli lasting for 7 s, but not after stimuli lasting for 1 s. Off-responses to the short stimuli appeared in a similar cortical region, when conditioning tonal stimuli lasting for 5–20 s preceded the stimuli. MK-801, an inhibitor of NMDA receptors, suppressed the two types of off-responses, suggesting that disinhibition produced by NMDA receptor-dependent synaptic depression might be involved in the off-responses. The peak off-responses were localized in a small region adjacent to the primary auditory cortex, and no frequency-dependent shift of the response peaks was found. Frequency matching of preceding tonal stimuli with short test stimuli was not required for inducing off-responses to short stimuli. Two-photon calcium imaging demonstrated significantly larger neuronal off-responses to stimuli lasting for 7 s in this field, compared with off-responses to stimuli lasting for 1 s. The present results indicate the presence of an auditory cortical field responding to long-lasting tonal offsets, possibly for temporal information processing.

Mice have been widely used in many recent studies on the auditory cortex, because of numerous available experimental techniques. Temporal information processing is important for auditory perception in mice, because species-specific vocalization of mice has complex temporal structures^1^,^2^. The auditory cortex plays important roles for discriminating temporal patterns in tonal stimuli^3^,^4^,^5^. Perception of tonal offsets or duration is one of the fundamental functions in temporal information processing, and neurons in the auditory cortex respond to offset of tonal stimuli lasting for a few seconds or less^6^,^7^,^8^,^9^,^10^. Off-responses are mainly produced by two different mechanisms: rebound excitation after synaptic inhibition^6^,^8^, and synaptic inputs coding tonal offsets produced in the lower levels of auditory neurons^9^,^10^. In addition to these mechanisms, cortical offset responses are likely modulated in a range longer than a few seconds, because the neural circuits of the auditory cortex exhibit various types of synaptic plasticity^11^,^12^,^13^. Properties of a particular neuron in the auditory cortex are strongly dependent on the cortical field of the neuron, and identification of cortical fields coding tonal offsets or duration is a crucial step for understanding the temporal information processing performed in the auditory cortex.

Previous electrophysiological studies have revealed that the mouse auditory cortex is composed of the core region of the primary auditory cortex (A1) and the anterior auditory field (AAF), and these two areas are surrounded by the belt region^14^,^15^. A1 and AAF are characterized by their tonotopically-organized maps, and the presence of a unique tonotopic map helps to identify a particular cortical field^16^. Since the accuracy of tonotopic maps obtained using extracellular recording is not sufficient, the classical maps in the auditory cortex have been elaborated by recent optical imaging studies using intrinsic hemoglobin signals^16^, endogenous flavoprotein fluorescence signals^17^,^18^,^19^,^20^, voltage-sensitive dyes^21^, and GFP-based calcium sensors^22^. At present, there are at least 7 independent cortical fields including A1, AAF, secondary auditory cortex (A2), the dorsoposterior (DP), dorsomedial (DM), dorsoanterior (DA), and the insular auditory field (IAF). Of these, DP and DA have no tonotopic structure, but are selectively activated by directional changes in frequency-modulated (FM) tones^17^,^18^,^19^,^20^. So far, identification of an auditory cortical field is based on properties of neuronal responses elicited at the onset of tonal stimuli. We previously recorded flavoprotein fluorescence responses to short tonal stimuli lasting for 0.5–1 s in the mouse auditory cortex, but clear off-responses were not found at the offset of such short stimuli^17^,^18^,^19^,^20^,^23^,^24^, probably because hemodynamic responses elicited approximately 1 s after stimulus onset interfere the later phase of responses^25^. In the present study, however, marked off-responses were observed at the offsets of long tonal stimuli lasting for 7 s. The peak off-responses were localized in a small cortical field between A1 and DP, and the same field was also activated by offsets of short tonal stimuli lasting for 1 s, when the short stimuli were preceded by conditioning tonal stimuli. Using two-photon calcium imaging of neuronal responses, we confirmed that neurons in the off-response field showed significantly larger off-responses to tonal stimuli lasting for 1 s compared with off-responses to stimuli lasting for 1 s. These findings suggest the presence of an auditory cortical field coding long-lasting tonal offsets or tonal duration in mice.

## Results

### Flavoprotein fluorescence responses at the offset of long-lasting tonal stimuli

Endogenous green fluorescence derived from mitochondrial flavoproteins reflects activity-dependent changes in aerobic energy metabolism in the brain, and is proportional to neural responses^26^,^27^,^28^. The functional identification of auditory cortical areas using flavoprotein fluorescence imaging in mice anesthetized with urethane is highly correlated with histochemical identification of cortical areas and thalamocortical projection patterns^19^,^20^. Therefore, we identified auditory cortical areas using this technique.

**Figure 1:**
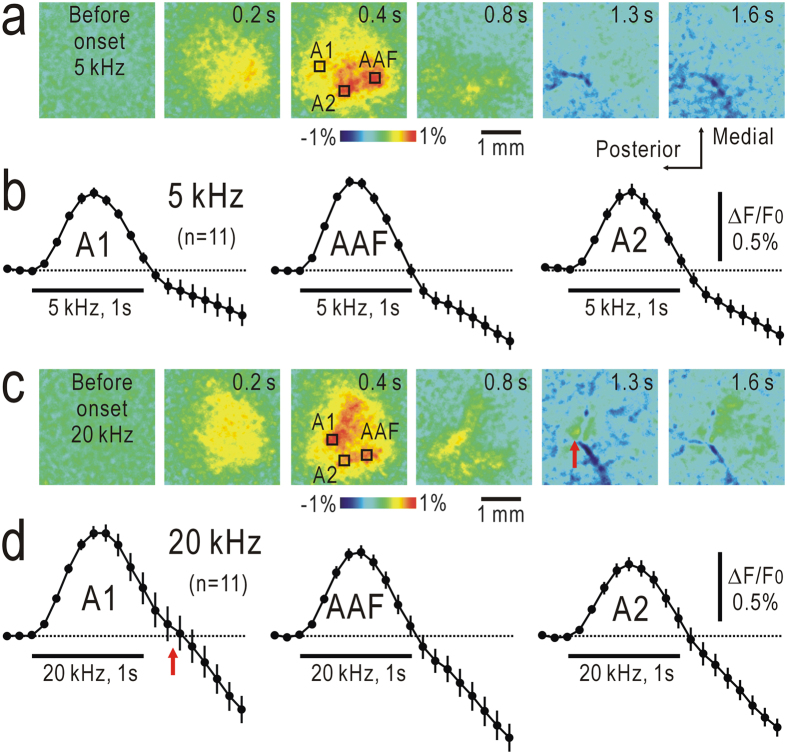
Flavoprotein fluorescence responses to tonal stimuli lsting for 1 s. (**a**) Responses to 5 kHz tone bursts. Maximal responses were found at approximately 0.4–0.5 s after the onset of the stimuli. Time shown at each panel represents that after stimulus onset. (**b**) Time courses of fluorescence signals in the ROI placed in A1, AAF, and A2 shown in (**a**). (**c**) Responses to 20 kHz tone bursts. (**d**) Time courses of fluorescence signals shown in (**c**). Humps (red arrows) were found in (**c**,**d**) after offset of stimuli. Mean and S.E.M. are shown.

When tonal stimuli at 5 kHz or 20 kHz lasting for 1 s were administered to mice, flavoprotein fluorescence responses were observed in A1, AAF, and A2 (Fig. 1a), as previously reported^17^,^18^,^19^,^20^,^23^. On-responses were measured as fractional fluorescence changes (ΔF/F_0_), in which F_0_ was recorded immediately before stimulus onset and ΔF was recorded after stimulus onset. The fluorescence increases reached to a peak at approximately 0.4–0.5 s after stimulus onset, while no apparent peak was measured at 0.4–0.5 s after the stimulus offset (Fig. 1b). When 20 kHz tonal stimuli were used, however, a slight hump (red arrows in Fig. 1c,d) was observed in the falling phase of the fluorescence responses measured in the region of the interest (ROI; 10 × 10 pixels) placed in A1. These results indicated that off-responses reported in previous studies^6^,^7^,^8^,^9^,^10^ are not sufficiently marked to produce an independent peak under our experimental conditions.

**Figure 2:**
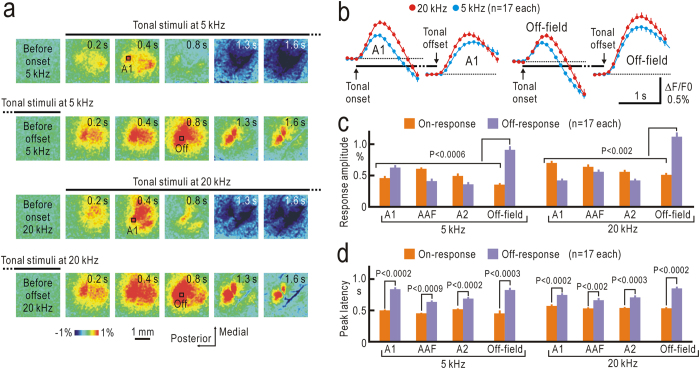
Flavoprotein fluorescence responses to tonal stimuli lasting for 7 s. (**a**) On- and off-responses to 5 kHz (upper two panels) and 20 kHz stimuli (lower two panels). Clear off-responses were observed after offsets of stimuli. Time shown at each panel represents that after stimulus onset for on-responses (first and third lines of panels), and that after stimulus offset for off-responses (second and fourth lines). Lines above panels show timing of stimulus presentation. (**b**) Time courses of on- and off-responses in ROIs placed in A1 and the off-response field. (**c**,**d**) Amplitudes (**c**) and peak latency (**d**) of on- and off-responses in A1, AAF, A2, and the off-response field.

Off-responses should be measured as fractional fluorescence changes (ΔF/F_0_), in which F_0_ is recorded immediately before stimulus offset and ΔF is recorded after stimulus offset. Estimation of off-responses, therefore, is affected by on-going on-responses and activity-dependent hemodynamic responses that follow on-responses^25^. To avoid this difficulty, the duration of tonal stimuli was extended to 7 s to obtain steady state fluorescence levels immediately before offset of stimuli. The time course of the on-responses to tonal stimuli lasting for 7 s was quite similar to that of the responses to tonal stimuli lasting for 1 s (Fig. 2a,b). However, the off-responses measured under this protocol were as marked as the on-responses (Fig. 2a,b). Quantitative measurement of response amplitudes revealed that off-responses were recorded in most of the auditory cortex including A1, AAF, and A2 (Fig. 2c). However, peak amplitudes of off-responses observed in a small cortical area, defined as off-response field, were significantly larger than any on- and off responses in A1, AAF, and A2 (P < 0.0006 for responses to tonal stimuli at 5 kHz, and P < 0.002 for responses at 20 kHz). The peak latency of off-responses was significantly larger than that of on-responses (P < 0.0002~0.002, Fig. 2d).

Off-responses were also observed after tonal stimuli at 10 kHz and 30 kHz lasting for 7 s (Supplementary Fig. S1a,b), and the results were similar to those obtained using 5 kHz and 20 kHz stimuli. However, no clear off-response was observed after white noise lasting for 7 s (Supplementary Fig. S1c), probably because afferent inputs activated by white noise at a particular frequency were not strong enough. When the duration of 20 kHz stimuli was changed, off-responses were observed in the range between 5 and 9 s, at least (Supplementary Fig. S1d).

### Lack of tonotopy in the off-response field

**Figure 3:**
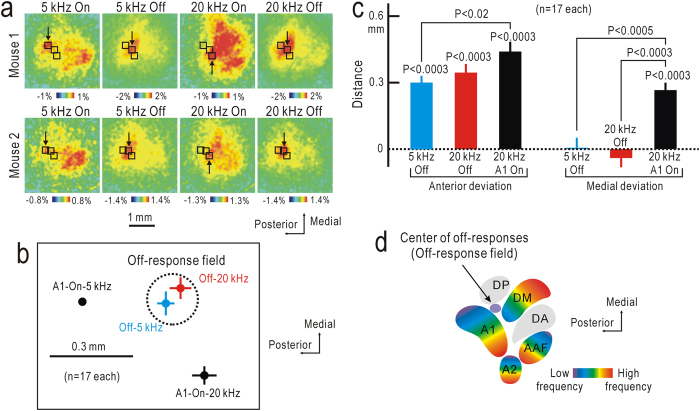
Cortical distribution of off-response peaks. (**a**) On- and off-responses to 5 kHz or 20 kHz tonal stimuli lasting for 7 s recorded in the two different mice. (**b**) Locations of on- and off response peaks relative to on-response peaks to 5 kHz stimuli in A1. No significant tonotopic shift was found in the off-responses, while the on-response peaks were arranged in a tonotopic order within A1. (**c**) Anterior and medial deviations of 5 kHz off-, 20 kHz off-, and 20 kHz on-response peaks from the 5 kHz on-response peaks in A1. *P* values were estimated compared with the location of the 5 kHz on-response peaks in A1, unless otherwise specified. (**d**) Relative location of off-response field (purple) in the schematic map of the auditory cortex^19^,^20^. Tonotopically organized fields are shown in rainbow colors.

Although the off-responses were found in most of the auditory cortex, the peaks seemed to be limited only in the off-response field located anterior and lateral to 5 kHz on-response peaks in A1 (Fig. 3a). We investigated the precise location of peak off-responses relative to peak on-responses to 5 kHz in A1. Peak off-responses to 5 or 20 kHz stimuli were observed only in a small cortical region (Fig. 3b). No significant difference was found in the location of peak off-responses to 5 or 20 kHz stimuli (Fig. 3c); therefore, no tonotopy seemed be present in the off-response field. In contrast, peak on-responses were tonotopically arranged, as previously reported^17^,^18^,^19^,^20^,^23^. The off-response field corresponded to a small area between A1 and DP in a schematic map of the auditory cortex^19^,^20^ (Fig. 3d).

### Off-responses to short test stimuli observed after conditioning stimuli

**Figure 4:**
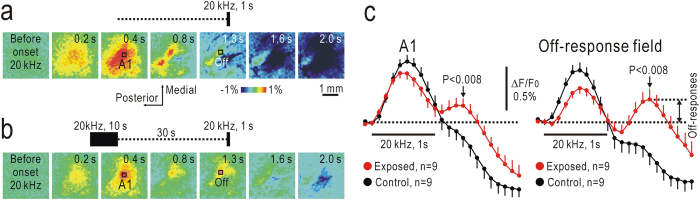
Off-responses to short test stimuli after conditioning stimuli. (**a**) Serial images of on-responses to short test stimuli at 20 kHz lasting for 1 s. Time shown at each panel represents that after stimulus onset. (**b**) Serial images of on- and off-responses to short test stimuli at 20 kHz for 1 s, which were given to mice 30 s after conditioning stimuli at 20 kHz for 10 s. Images in (**a**,**b**) were taken in the same mouse. (**c**) Time courses of ΔF/F_0_ in ROIs placed at on-response peaks in A1 and off-response peaks in the off-response field shown in (**a**,**b**) with or without preceding conditioning stimuli. Amplitudes of off-responses to short test stimuli were defined as ΔF/F_0_. When this value was negative, the amplitude was assumed to be zero.

The off-responses after long tonal stimuli lasting for 7 were clearly observed as ΔF/F_0_, partly because F_0_ was obtained from stabilized fluorescence levels immediately before offset of tonal stimuli. However, exposure to long-lasting stimuli may also modulate the off-responses by various types of synaptic plasticity in neural circuits of the auditory cortex^11^,^12^,^13^. To test this latter possibility, mice were exposed to conditioning tonal stimuli lasting for 10 s. Short test stimuli lasting for 1 s were given to the mice 30 s after conditioning stimuli (Fig. 4a,b). The on-responses were slightly depressed after the conditioning stimuli compared with those without the preceding stimuli (Fig. 4c), but these changes were not statistically significant. In contrast, off-responses were clearly observed after offset of short test stimuli (Fig. 4a,b), and distinct peaks were found in traces of ΔF/F_0_ changes (Fig. 4c). Values in ΔF/F_0_ at the peaks were significantly larger in A1 and the off-response field than those recorded without preceding stimuli (P < 0.008 for both). These results indicated that off-responses were selectively enhanced by preceding conditioning stimuli.

**Figure 5:**
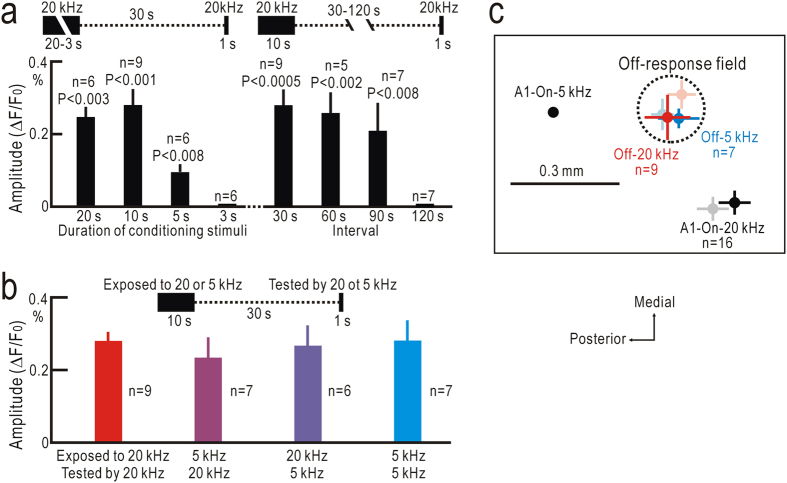
Properties of off-responses to short test stimuli. (**a**) Amplitudes of off-responses dependent on duration of conditioning stimuli (left) and intervals between conditioning and test stimuli (right). Statistical differences were evaluated compared with response amplitudes at the 3 s duration of conditioning stimuli (left), or at the 120 s interval between conditioning and test stimuli (right). (**b**) Relationship between amplitudes of off-responses, and frequencies of conditioning and test stimuli. No apparent frequency specificity was found. (**c**) Cortical locations of off-response peaks relative to on-response peaks to 5 kHz stimuli in A1. Off-response peaks after conditioning stimuli (vivid colors) were observed in the same area as the response peaks (faint colors) found after long sound stimuli lasting for 7 s.

Properties of off-responses to short test stimuli were investigated. We modified the parameters of the preceding conditioning stimuli. The effect of conditioning stimuli lasting for 10 s was similar to that of stimuli lasting for 20 s (Fig. 5a, left). Although still significant, the stimuli lasting for 5 s were less effective compared with stimuli lasting for 10 s. The stimuli lasting for 3 s were not effective. We further changed the intervals between preceding conditioning stimuli lasting for 10 s and the short test stimuli (Fig. 5a, right). The conditioning stimuli 60 s before the short test stimuli were as effective as the conditioning stimuli 30 s before the test stimuli. Although still significant, the conditioning stimuli were less effective at an internal of 90 s, and it was not effective at 120 s. Next, we changed frequencies of the conditioning stimuli and test stimuli at 5 kHz or 20 kHz, independently (Fig. 5b). However, no apparent frequency specificity was found regarding amplitudes of off-responses. The off-response peaks after conditioning stimuli were observed at almost the same places (circles and bars with vivid colors in Fig. 5c) of off-responses observed after long tonal stimuli lasting for 7 s (circles and bars with faint colors in Fig. 5c), suggesting that both types of off-responses share underlying mechanisms. It is also suggested that some types of short-term synaptic plasticity may be the mechanisms enhancing the off-responses, since the effects of conditioning stimuli were maintained for 90 s with no activity during this period.

### Roles of NMDA receptors

**Figure 6:**
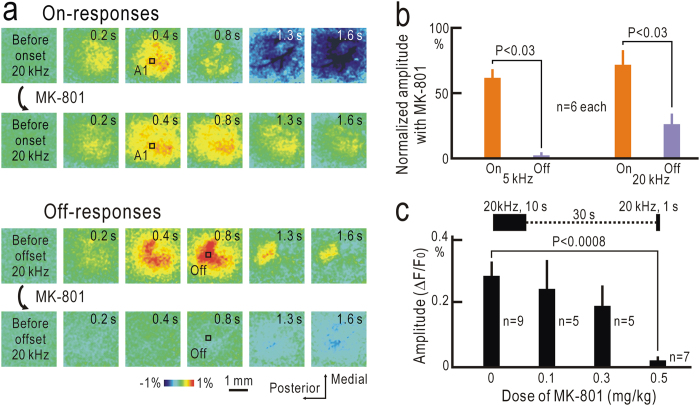
Suppression of off-responses by MK-801. (**a**) On- and off responses to 20 kHz tonal stimuli lasting for 7 s recorded before and after application of MK-801 (0.5 mg/kg, i.p.) in the same mouse. Time shown at each panel represents that after stimulus onset for on-responses (first and second lines of panels), and that after stimulus offset for off-responses (third and fourth lines). (**b**) Relative amplitudes of on- responses measured in A1 and off-responses measured in the off-response field to 5 kHz or 20 kHz tonal stimuli after application of 0.5 mg/kg MK-801. Values were normalized by the amplitudes before application. (**c**) Relationship between MK-801 dose and amplitudes of off-responses in the off-response field to short test stimuli at 20 kHz.

The long-lasting effects of conditioning stimuli are likely mediated by short-term plasticity. Such short-term plasticity in the auditory cortex has important roles in auditory information processing^29^,^30^. Because many types of synaptic plasticity are dependent on NMDA receptors^31^,^32^,^33^,^34^,^35^,^36^, we tested the effects of MK-801, a blocker of NMDA receptors^37^, on off-responses observed after sound stimuli lasting for 7 s (Fig. 6a,b). On-responses were modestly suppressed by MK-801, probably due to the suppressive effect on excitatory synaptic transmission partly mediated by NMDA receptors. However, MK-801 more clearly suppressed the off-responses at a dose of 0.5 mg/kg (i.p.), while MK-801 at a dose of 0.5 mg/kg was also effective on off-responses to short test stimuli, but not at a dose of 0.1 or 0.3 mg/kg (Fig. 6c). These results strongly suggested that NMDA receptor-dependent short-term plasticity was required for off-responses to long tonal stimuli, or those after conditioning stimuli.

### Two-photon calcium imaging of neural activities in the off-response field

**Figure 7:**
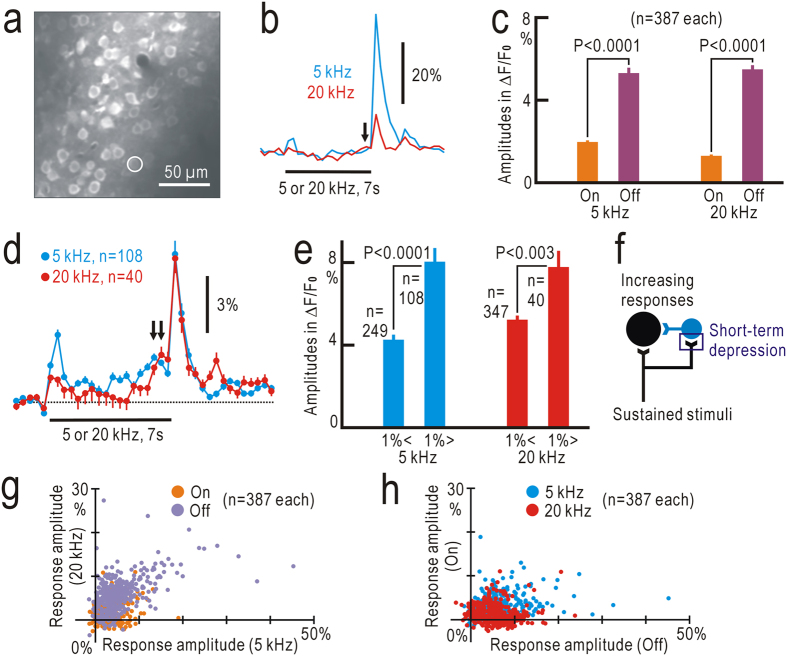
Neuronal calcium responses to tonal stimuli recorded at 397 ms intervals. (**a**) Two-photon calcium imaging of the off-response field stained with Cal-520. The circle with a diameter of 20 pixels (14.3 μm) shows a neuronal cell body that was stained with Cal-520, but not with SR-101. (**b**) Example of calcium responses in ΔF/F_0_ to 5 kHz and 20 kHz stimuli lasting for 7 s, recorded in the cell body shown in (**a**). Arrow shows gradually-increasing responses before offset of tonal stimuli. The reference (F_0_) was taken immediately before stimulus onset, because the Ca^2 + ^signals of relatively large amplitudes were less clearly affected by hemodynamic responses. (**c**) Amplitudes of the on- and off-responses to 5 kHz and 20 kHz stimuli. (**d**) Averaged traces of ΔF/F_0_ in selected neurons, which exhibited ΔF/F_0_ larger than 1% in three frames immediately before tonal offset. Double arrows show increasing responses before tonal offset. (**e**) Amplitudes of off-responses to 5 kHz and 20 kHz stimuli in neurons with increasing responses >1% or <1%. (**f**) Possible disinhibition that produces increasing responses and enhances off-responses after long-lasting tonal stimuli. An inhibitory neuron is shown in blue. (**g**) Correlation between responses elicited by 5 kHz and 20 kHz stimuli. Orange and purple dots show on- and off-responses, respectively. (**h**) Correlation between on- and off-responses. Blue and red dots show data obtained using 5 kHz and 20 kHz stimuli, respectively.

Recent studies using two-photon imaging of neuronal calcium responses have disclosed physiological properties of neurons in the auditory cortex^17^,^19^,^22^,^38^,^39^. Because the results obtained by flavoprotein fluorescence imaging suggested the presence of neurons specified for tonal offset, we tested this hypothesis using two-photon calcium imaging recorded at 397 ms intervals. First, the off-response field was identified using flavoprotein fluorescence imaging. We identified 387 neuronal cell bodies stained with Cal-520, a fluorescence calcium indicater^40^, in the off-response field of three mice (Fig. 7a). Some of these neurons exhibited both on- and off-responses to 5 kHz and 20 kHz stimuli (Fig. 7b). Amplitudes of off-responses elicited by 5 kHz and 20 kHz tonal stimuli were significantly larger than those of the corresponding on-responses, respectively (P < 0.0001 for each, Fig. 7c). The sample traces in Fig. 7b show that gradually-increasing responses preceded the tonal offset (single arrow in Fig. 7b). The increasing responses were clearly observed in the selected neurons, which exhibited ΔF/F_0_ larger than 1% in three frames immediately before the tonal offset (Fig. 7d, double arrows). Amplitudes of the off-responses were significantly larger in neurons with increasing responses >1% compared with those in other neurons with increasing responses <1% (P < 0.0001 for off responses to 5 kHz stimuli, and P < 0.003 for 20 Hz stimuli Fig. 7e). The increasing responses cannot be explained as rebound activities, because rebound activities are not induced during stimulus presentation. On the other hand, the increasing responses might reflect disinhibition elicited by sustained tonal stimuli (Fig. 7f). The abrupt Ca^2+^ increases immediately after stimulus offset can be also explained by disinhibition but not by residual on-responses. There was a significant correlation between response amplitudes to 5 kHz and 20 kHz stimuli (correlation coefficient: 0.56, P < 0.0001, Fig. 7g). In contrast, the correlation between on- and off-response amplitudes was weak, although significant (correlation coefficient: 0.19, P < 0.0001, Fig. 7h).

Two-photon calcium imaging with a shorter temporal resolution can be an alternative to flavoprotein fluorescence imaging. Using a temporal resolution of 140 ms, we separated on- and off-responses recorded to tonal stimuli lasting for 1 s in 44 neurons (Supplementary Fig. S2). It was possible probably due to fast time courses of Ca^2+^ responses compared with those of flavoprotein fluorescence responses triggered by Ca^2+^ responses. We also confirmed that the off-responses to stimuli lasting for 7 s were significantly larger than those to stimuli lasting for 1 s in the same neurons (P < 0.0001). These results indicate that essentially similar results were obtained using the two different methods, although off-responses to short tonal stimuli could be clearly resolved only by two-photon calcium imaging recorded at 140 ms intervals.

## Discussion

### Auditory cortical fields specific to temporal stimulus patterns in mice

The auditory cortex is divided into various fields of distinct response properties; therefore, identification of cortical fields is an important step for understanding the roles of the auditory cortex. Recently, optical imaging revealed the presence of many fields in the mouse auditory cortex. In addition to classical A1, AAF, and A2 identified using electrophysiological recordings^14^,^15^, DM^19^,^20^ and IAF^21^,^41^ were found as tonotopically-organized fields. DP and DA (previously named as UF) were also identified as regions specifically activated by directional reversal of FM sounds with no apparent tonotopy^17^,^18^,^19^,^20^. In the present study, we focused on off-responses to delineate a new region, because analysis of temporal information included in tonal stimuli is an essential function of the auditory cortex^3^,^4^,^5^. Although off-responses have been widely observed in the auditory cortex^6^,^7^,^8^,^9^,^10^, quantitative analysis of peak off-response distribution revealed the presence of a localized off-response specific field between A1 and DP. In contrast, neurons in the visual cortex also respond to the onsets and offsets of stimuli^42^,^43^, and cortical distribution of visual on-responses is essentially similar to that of visual off-responses when investigated using flavoprotein fluorescence imaging in mice^27^. The present results in the auditory cortex, however, are compatible with a previous electrophysiological study; on- and off-responses were reported to distribute differently in rats^8^. Because we used endogenous fluoresce signals that homogenously distribute and quantitatively reflect neuronal activity^26^,^27^,^28^, the presence of the off-response field is unlikely an artifact of heterogeneous distribution of fluorophores used for imaging experiments. Furthermore, two-photon calcium imaging of neuronal activities in this region confirmed that neurons exhibited more marked off-responses compared with on-responses in the same neurons. The off-response field with no apparent tonotopic map is clearly different from A1, which is tonotopically organized. The off-response field specified for off-responses is also clearly different from DP, which is specifically activated by directional changes in FM sounds^17^,^18^,^19^,^20^. Taken together, it is concluded that the off-response field is the auditory region specific to temporal patterns of tonal stimuli and has been identified for the first time in mice.

### Gating mechanisms for cortical off-responses

Responses to offset of tonal stimuli have been recorded in various levels of the auditory system including the cochlear nucleus^44^, superior olivary nuclei^45^,^46^, nuclei of lateral lemniscus^47^, inferior colliculus^48^,^49^, and medial geniculate nuclei^50^, as well as the auditory cortex^6^,^7^,^8^,^9^,^10^. Off-responses are thought to be generated by two mechanisms: rebound excitation after long-lasting synaptic inhibition^6^,^8^, and synaptic inputs coding tonal offsets detected in the lower levels of auditory neurons^9^,^10^. We found that off-responses were enhanced after tonal stimuli lasting for 7 s, which was longer than stimuli used in previous studies. Clear off-responses were also observed at the offset of short tonal stimuli for 1 s, when preceding conditioning stimuli were given to mice. Because the effect of preceding conditioning stimuli was maintained for at least 90 s, they must be mediated by short-term plasticity rather than rebound excitation after long-lasting synaptic inhibition. The suppression of off-responses by MK-801 suggests that short-term plasticity dependent on NMDA receptors plays an important role in off-responses. The short-term plasticity could be induced in any levels of the auditory system, because off-responses have been reported in subcortical neurons. However, the presence of a long-range gating mechanism lasting for at least 90 s has not been found at subcortical levels. Considering the sharply localized off-response field in the auditory cortex, off-responses in the present study are probably regulated and enhanced by short-term plasticity within cortical neural circuits.

Short-term plasticity is divided into short-term potentiation and short-term depression^51^. Of these, induction of cortical short-term potentiation in mice anesthetized with urethane requires high-frequency electrical stimulation^52^,^53^. Therefore, it is unlikely induced by natural tonal stimuli. In contrast, short-term depression is induced by low-frequency stimulation^13^,^54^, and may be induced by natural tonal stimuli lasting for several seconds. Consistent with this idea, on-responses to tonal stimuli were depressed by preceding conditioning stimuli (Fig. 4), although these changes were not significant. When short-term depression was induced in inhibitory neurons, disinhibition and enhancement of off-responses in nearby neurons was produced by the depression (Fig. 7f). Two-photon imaging revealed the presence of gradually increasing responses, which preceded tonal offset in some cortical neurons. The off-response amplitudes were larger in these neurons compared with those in other neurons without the gradually increasing responses. These findings are well explained if the gradually increasing responses reflected disinhibition induced by short-term depression in nearby inhibitory neurons. Other neurons with no apparent increasing response also exhibited off-responses, possibly via synaptic inputs from neurons with gradually increasing responses. A unique point of short-term depression is that it is maintained for several tens of seconds, which cannot be covered by other mechanisms, such as rebound excitation or long-lasting metabotropic receptors. Therefore, the temporal gating mechanism of off-responses in the auditory cortex may also play a role in other forms of temporal information processing in the range of several tens of seconds. When combined with heterosynaptic plasticity found in neural circuits of the auditory cortex^12^,^55^, these long-range gating mechanisms are expected to play important roles in temporal information processing in auditory perception. Another possibility is that preceding tonal stimuli may have some contextual effects on subsequent responses in the auditory cortex^56^,^57^, possibly through the same long-range mechanisms.

### Roles of the off-response field in auditory perception

Species-specific vocalization of mice has components with complex temporal structures^1^,^2^. Therefore, temporal information processing included in species-specific vocalization must be required for vocal communication in mice. The auditory cortex plays important roles in discriminating temporal patterns in tonal stimuli^3^,^4^,^5^. The present off-response field that codes long-lasting tonal offsets is a candidate for regions specialized in temporal information processing. Interestingly, no apparent tonotopy was found in the off-response field, and neurons responded to tonal offset without frequency specificity. This frequency-nonspecific temporal information is unlikely derived from other cortical fields: other regions in the auditory cortex responded to long tonal stimuli only transiently (for example, Fig. 1), although induction of NMDA receptor dependent short-term depression requires sustained synaptic inputs. These sustained inputs with tonal offset information may be derived from the medial geniculate nuclei^50^. The off-response field, therefore, is likely formed based on localized thalamic inputs to this region, such as other fields in the auditory cortex receiving their own thalamic inputs^17^,^18^,^19^,^20^,^58^,^59^. However, subcortical inputs can be converted to neuronal activities coding long-lasting tonal offsets within the neural circuits of the off-response filed in a frequency-independent manner. Furthermore, information processed in the off-response field may be integrated with other aspects of tonal stimuli somewhere in the brain. The wide distribution of off-responses in regions such as A1 and DP, adjacent to the off-response field, strongly suggest that the auditory cortex is the responsible region where temporal information is integrated again with other information such as tonal frequencies represented in A1 and directions of FM tones represented in DP, possibly for auditory perception in vocal communication.

## Materials and Methods

### Ethical approval and animals

The experiments in the present study were approved by the ethics committee of animal experiments in Niigata University (approved number: 233-4 and 372-7), and were carried out in accordance with the approved guidelines. Male C57BL/6 mice, between 5 and 7 weeks old purchased from Charles River Japan (Tokyo, Japan), were used in the present study.

### Transcranial flavoprotein fluorescence imaging

Tonal responses in the mouse auditory cortex were imaged, as described in our previous studies^4^,^6^. Mice were anesthetized with urethane (1.6 g/kg, i.p.). Spontaneous respiration of O_2_ gas was maintained, and rectal temperature was kept at 37.5 °C with a heating pad. After a subcutaneous injection of bupivacaine, a local anesthetic, the skin covering the skull was disinfected and incised. The temporal muscle over the auditory cortex was removed. A piece of metal was attached to the skull using dental resin, and the head was fixed in place by screwing the metal piece onto a manipulator. The exposed surface of the intact skull was covered with liquid paraffin to prevent drying and to keep the skull transparent. The operation was finished within 30 min. Imaging experiments were started approximately 1 h after introducing anesthesia and were usually finished within 3 h, during which conditions of anesthetized mice were stable. After the imaging experiments, mice were sacrificed with a pentobarbital overdose (300 mg/kg, i.p.).

Cortical images (128×168 pixels) of endogenous green fluorescence (λ = 500–550 nm) in blue light (λ = 450–490 nm) were recorded by a cooled CCD camera (ORCA-ER, Hamamatsu Photonics, Hamamatsu, Japan). Images were taken at 9 frames/s using a camera attached to a binocular epifluorescence microscope (M651 combined with MZ FL II, Leica Microsystems, Wetzlar, Germany). As tonal stimuli to elicit neural responses in the auditory cortex, sine waves at a frequency of 5 or 20 kHz with a 5 ms rise/fall time were applied through a speaker (SRS-3050A, Stax, Saitama, Japan) placed in front of the mice. The intensity was approximately 65 dB SPL. Images were taken in a recording session in trials repeated at 60 s intervals. When preceding conditioning stimuli were used, trials were repeated at 180 s intervals. The images elicited by a particular stimulus or stimulus condition were averaged over 24 trials. Spatial averaging of 5×5 pixels was used to improve image quality. Images were normalized, pixel by pixel, with respect to a reference image, which was obtained by averaging five images taken immediately before stimulus onsets or offsets. The normalized images were generated using a pseudocolor scale in terms of relative fluorescence changes (ΔF/F_0_). Auditory fields were defined as the regions producing >60% maximum response to tones, relative to baseline fluorescence level. These activity patterns were stable from mouse to mouse, and tonotopically arranged in the A1, AAF, A2 and DM (Fig. 3d). Square windows of 10×10 pixels (0.26×0.26 mm) in the figures (for example, Figs 1a,c and 2a) indicate the peak response to the stimulus within each field. The response amplitude in ΔF/F_0_ was evaluated in the windows.

When the role of NMDA receptors was investigated, imaging experiments were performed before and 30 min after intraperitoneal injection with MK-801 hydrogen maleate (0.1–0.5 mg/kg, Sigma Aldrich, St. Louis, USA) dissolved in saline.

### Two-photon calcium imaging in the off-response selective area

Prior to two-photon calcium imaging, the precise location of the off-response field was identified using flavoprotein fluorescence imaging. Two-photon calcium imaging was performed using Cal-520 as a calcium indicator^40^. The skull over the off-response field was removed using a dental drill. Cal-520 AM (AAT Bioquest, Sunnyvale, USA) at 8 mM was dissolved in dimethyl sulfoxide containing 20% (w/v) pluronic F-127 (Invitrogen, Carlsbad, USA), which is a surfactant, and further diluted 10 times with saline containing 0.1 mM sulforhodamine 101 (SR-101, Invitrogen). A glass pipette (tip diameter: 2–4 μm) was pulled and filled with the Cal-520 AM solution. The pipette was slowly inserted and advanced into the supragranular layers, 200–250 μm deep from the pial surface. The Cal-520 AM solution was injected with a pressure of 8–25 kPa for 5–10 min, so that the cells in the area 200–300 μm from the tip of the pipette were stained. Astrocytes stained with SR-101 were excluded from further analysis. After the injection, the pipette was withdrawn and the hole made by the craniotomy was covered with 2% agarose and a thin cover glass (5 × 5 mm, thickness <0.15 mm). The cover glass was firmly fixed to the skull with dental resin. Images were obtained via a ×20 water immersion objective lens (numerical aperture 1.0, HCX PL APO; Leica Microsystems). Calcium imaging was performed using a two-photon microscope (TCS SP5 MP, Leica Microsystems) with a Ti-Sapphire mode-locked femto second laser (Chameleon Vision, Coherent, Santa Clara, USA). The excitation wavelength for Cal-520 was 800 nm, while that for SR-101 was 900–950 nm. Observations were started 40–50 min after Cal-520 AM injection. Laser power was kept under 20 mW throughout the experiments. Cal-520 fluorescence was observed between 500 and 550 nm, and 256 × 256 pixels images were recorded at 397 ms intervals over an area 183 × 183 μm in size. Circular windows with a diameter of 20 pixels (14.3 μm) were placed on green neuronal cell bodies stained with Cal-520 but not with SR-101, and obtained fluorescence signals in the windows were averaged over 10 trials. The normalized fluorescence changes in ΔF/F_0_ were calculated using the averaged baseline intensity (F_0_) collected immediately before stimulus onset.

### Statistical analysis

Statistical significance was analyzed using StatView software (SAS Institute Inc., Cary, USA). Unpaired data were evaluated using the Mann-Whitney U test, while paired data were evaluated by the Wilcoxon signed rank test. Only *P* values < 0.05 are shown in the figures. Correlation coefficients and their significance were also evaluated.

## Additional Information

**How to cite this article**: Baba, H. *et al*. Auditory cortical field coding long-lasting tonal offsets in mice. *Sci. Rep.* **6**, 34421; doi: 10.1038/srep34421 (2016).

## Supplementary Material

Supplementary Information
