# Supplementary material for: Auditory cortical field coding long-lasting tonal offsets in mice
Source: Sci Rep. 2016 Sep 30;6:34421. doi: 10.1038/srep34421 (PMC5043382; doi:10.1038/srep34421)
Supplement: Supplementary Information [file srep34421-s1.pdf]

Supplementary Figures S1 and S2

Auditory cortical field coding long-lasting tonal offsets in mice

Hironori Baba[1, 2], Hiroaki Tsukano[1], Ryuichi Hishida[1], Kuniyuki Takahashi[2], Arata Horii[2], Sugata Takahashi[2], Katsuei Shibuki[1]

1. Department of Neurophysiology, Brain Research Institute, Niigata University, 1-757 Asahi-machi, Chuo-ku, Niigata 951-8585, Japan
2. Department of Otolaryngology, Head and Neck Surgery, Graduate School of Medical and Dental Sciences, Niigata University, 1-757 Asahi-machi, Chuo-ku, Niigata 951-8510, Japan

Correspondence: Katsuei Shibuki

Department of Neurophysiology, Brain Research Institute, Niigata University

1-757 Asahi-machi, Chuo-ku, Niigata 951-8585, Japan

E-mail: shibuki@bri.niigata-u.ac.jp; Phone: +81-25-227-0625; Fax: +81-25-227-0628

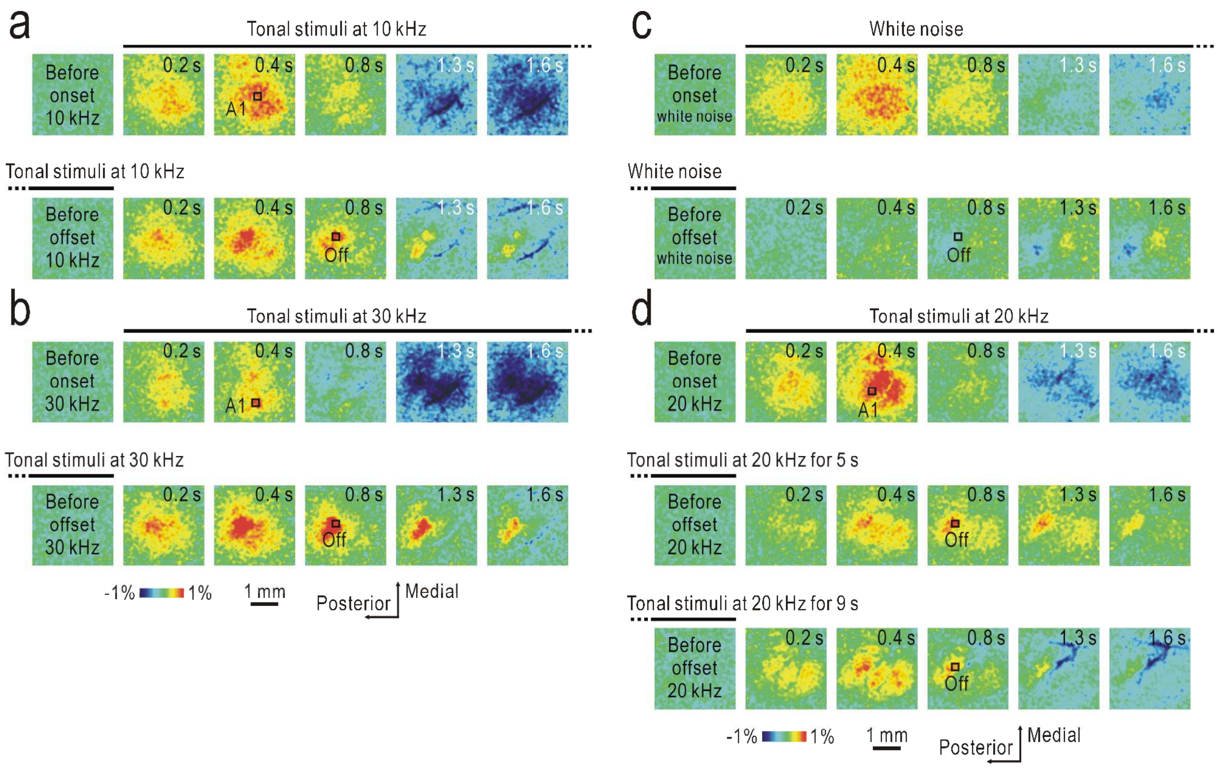

Supplementary Figure S1. On- and off-responses to tonal stimuli recorded by flavoprotein fluorescence imaging.

a, On- and off-responses to 10 kHz stimuli lasting for 7 s. **b,** On- and off-responses to 30 kHz stimuli lasting for 7 s. **c,** On-response to white noise lasting for 7 s. No apparent off-response was found. Responses in **a**, **b** and **c** were obtained from the same mouse. Similar results were obtained in 4 mice. ROIs of 10×10 pixels show the places where $\Delta F/F_0$ was maximal in A1 or the off-response field. **d,** On- and off-responses to 20 Hz stimuli lasting for 5 s and 9 s.

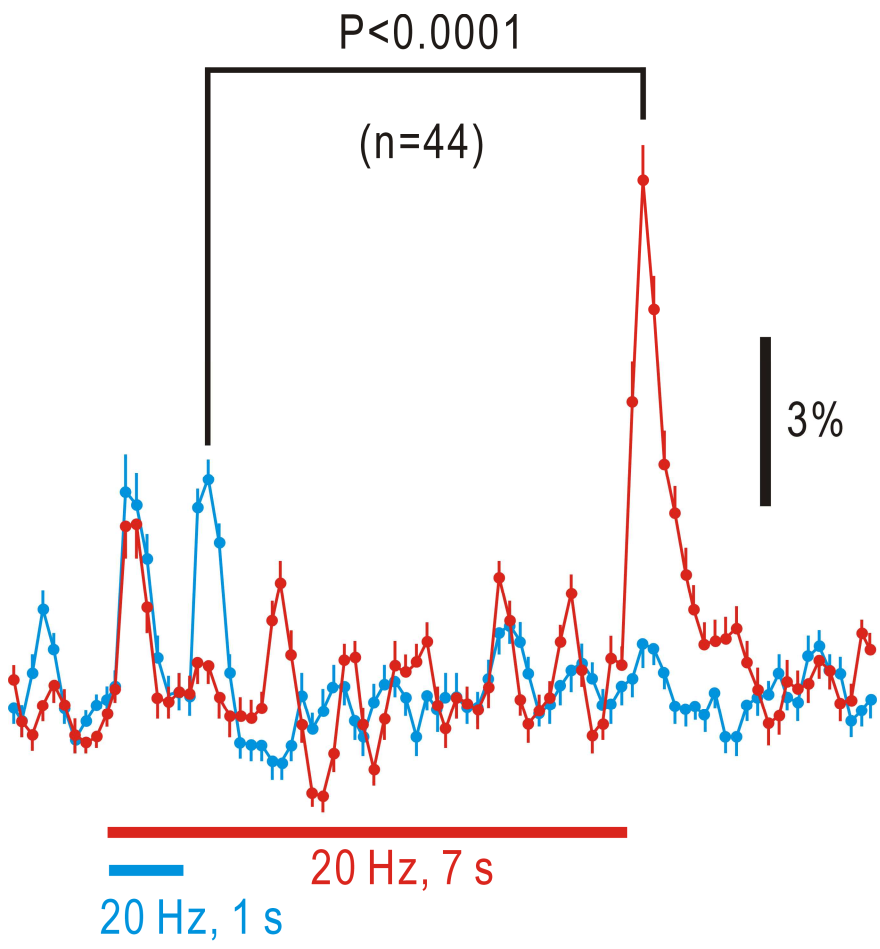

Supplementary Figure S2. Two-photon calcium responses to tonal stimuli recorded at 140 ms intervals.

Averaged traces (mean and S.E.M.) of $\Delta F/F_0$ in 44 neurons stained with Cal-520. The red trace represents responses to 20 kHz stimuli lasting for 7s, and the blue trace shows responses to 20 kHz stimuli lasting for 1s in the same neurons. The off-responses to stimuli lasting for 7 s were significantly larger than the on-responses to stimuli lasting for 1 s (P<0.0001).
